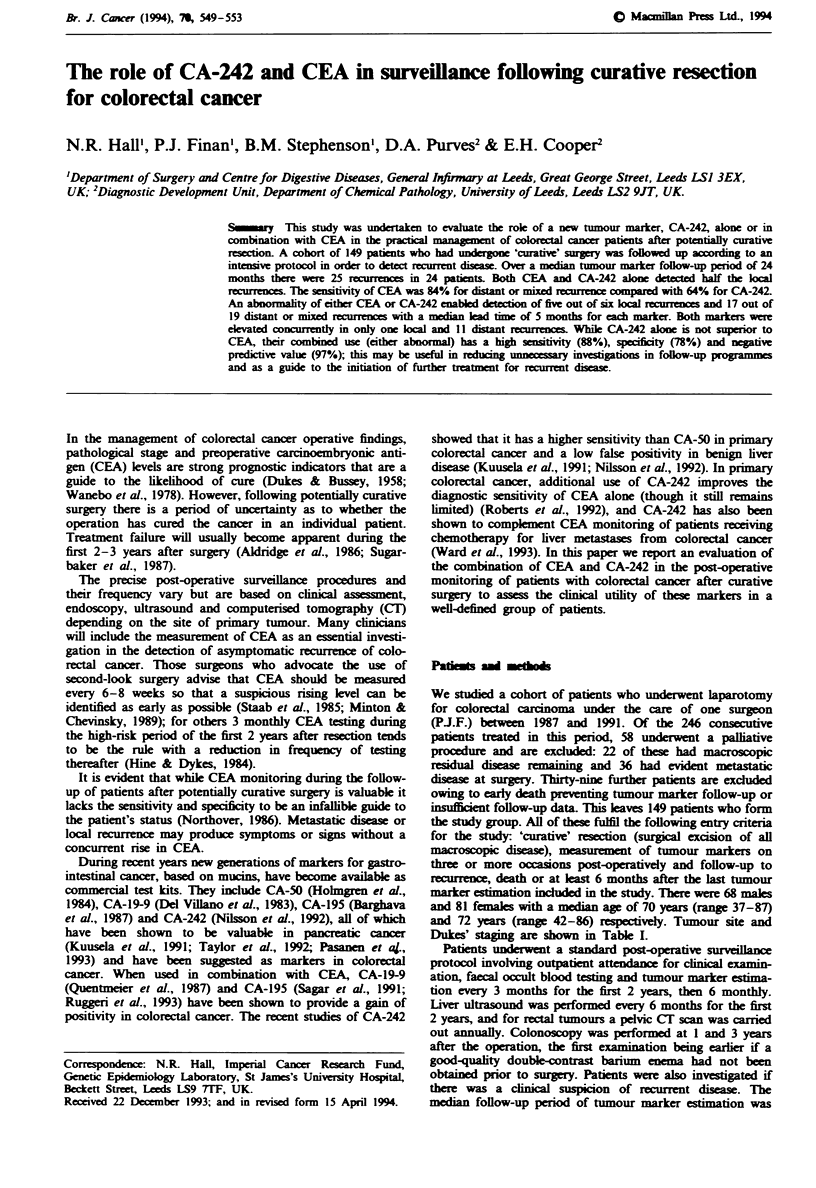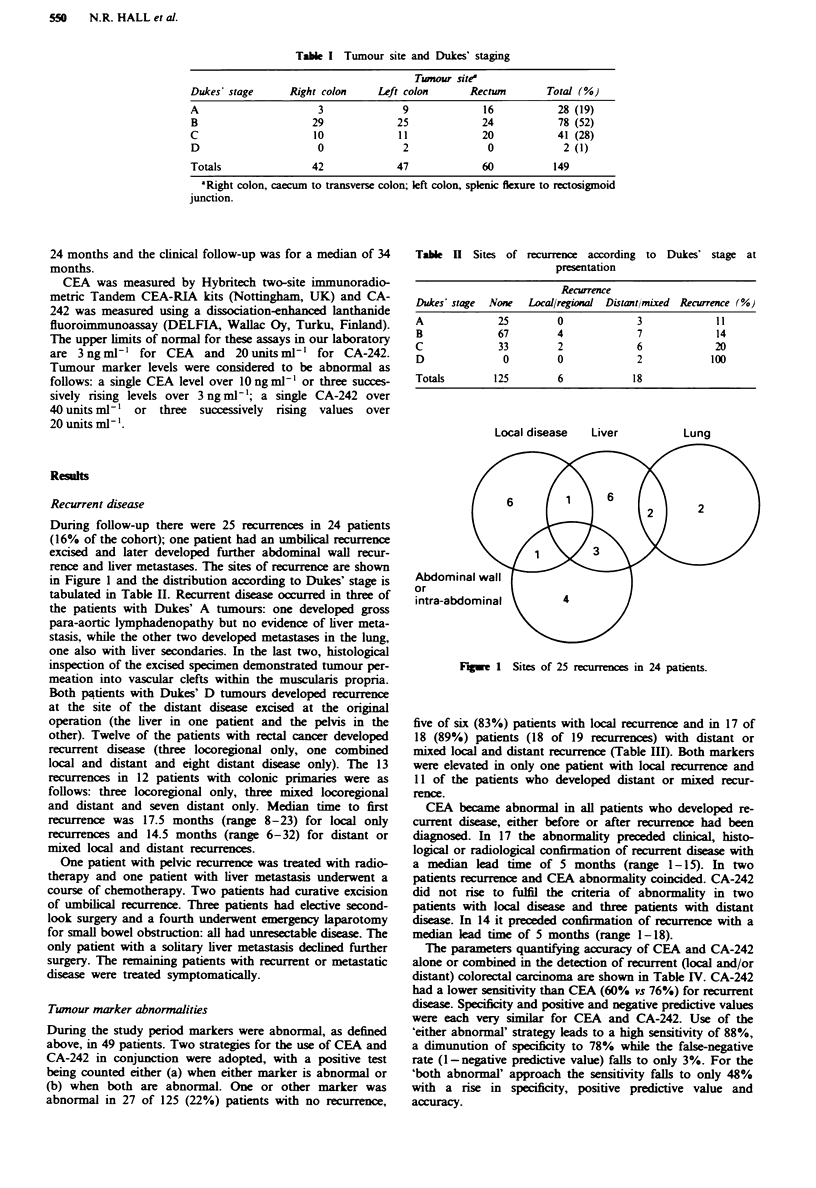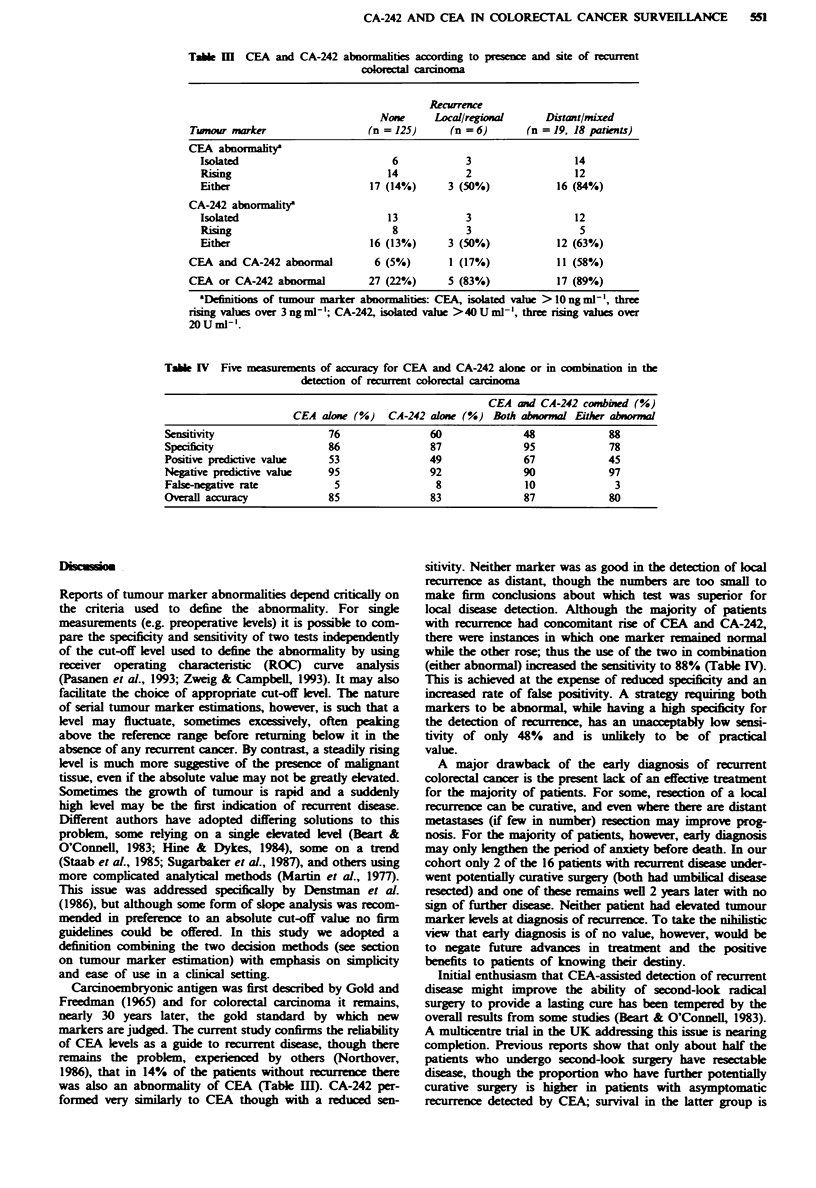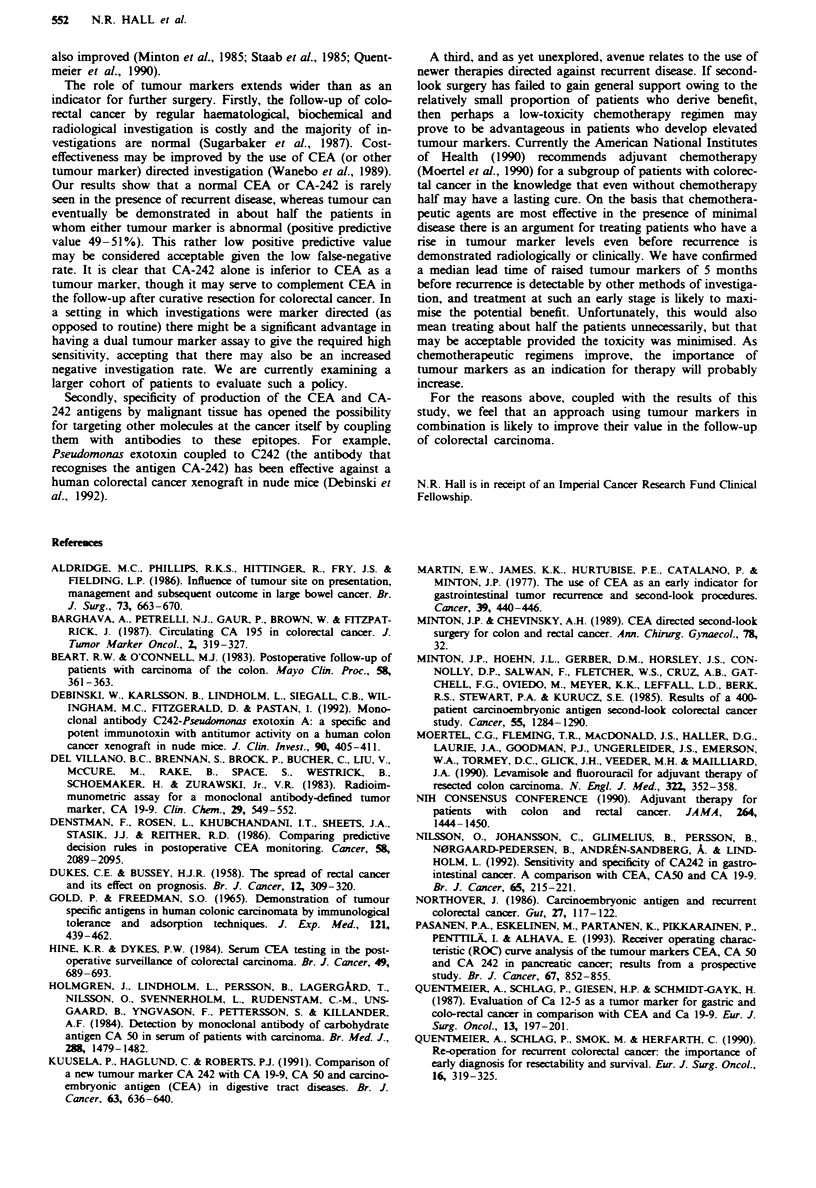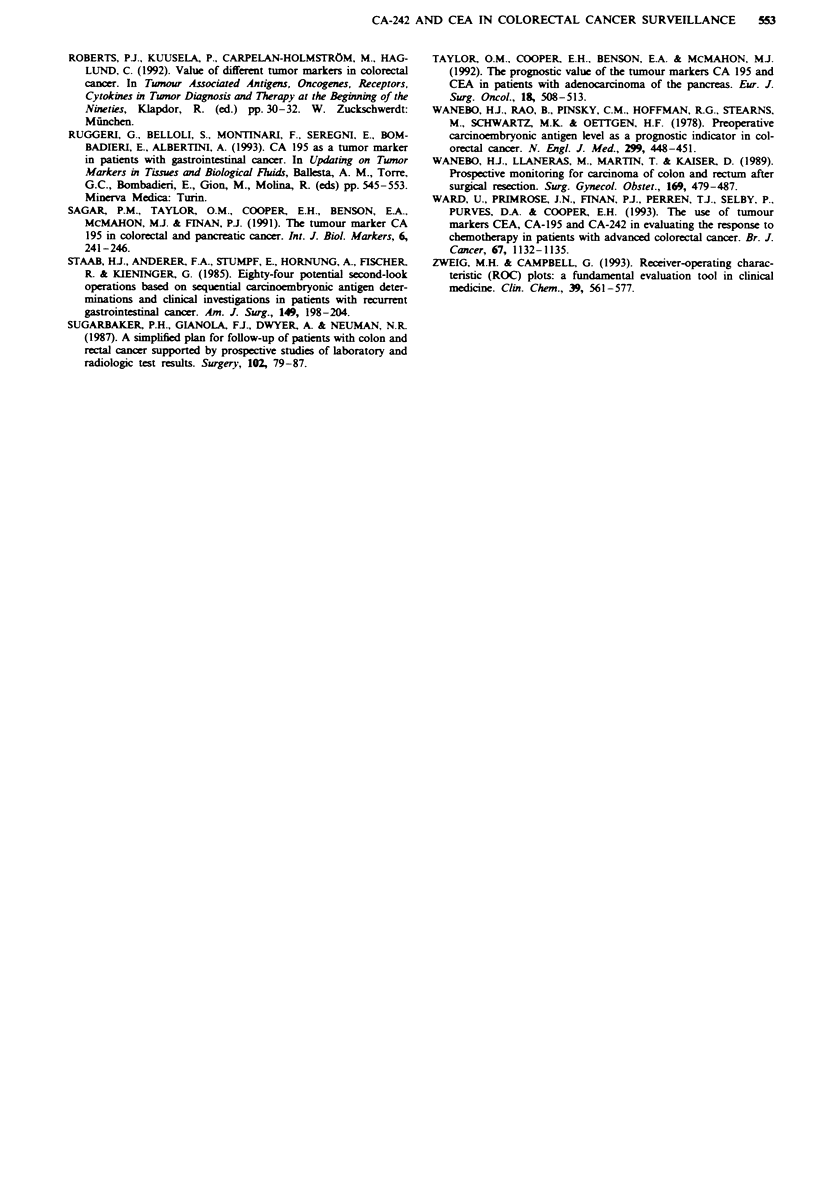# The role of CA-242 and CEA in surveillance following curative resection for colorectal cancer.

**DOI:** 10.1038/bjc.1994.343

**Published:** 1994-09

**Authors:** N. R. Hall, P. J. Finan, B. M. Stephenson, D. A. Purves, E. H. Cooper

**Affiliations:** Department of Surgery, General Infirmary at Leeds, UK.

## Abstract

This study was undertaken to evaluate the role of a new tumour marker, CA-242, alone or in combination with CEA in the practical management of colorectal cancer patients after potentially curative resection. A cohort of 149 patients who had undergone 'curative' surgery was followed up according to an intensive protocol in order to detect recurrent disease. Over a median tumour marker follow-up period of 24 months there were 25 recurrences in 24 patients. Both CEA and CA-242 alone detected half the local recurrences. The sensitivity of CEA was 84% for distant or mixed recurrence compared with 64% for CA-242. An abnormality of either CEA or CA-242 enabled detection of five out of six local recurrences and 17 out of 19 distant or mixed recurrences with a median lead time of 5 months for each marker. Both markers were elevated concurrently in only one local and 11 distant recurrences. While CA-242 alone is not superior to CEA, their combined use (either abnormal) has a high sensitivity (88%), specificity (78%) and negative predictive value (97%); this may be useful in reducing unnecessary investigations in follow-up programmes and as a guide to the initiation of further treatment for recurrent disease.


Br. J. Cancer (1994), 70, 549-553 © Macmillan Press Ltd., 1994

### The role of CA-242 and CEA in surveillance following curative resection for colorectal cancer

N.R. Hall[1], P.J. Finan[1], B.M. Stephenson[1], D.A. Purves[2] & E.H. Cooper[2]

[1]*Department of Surgery and Centre for Digestive Diseases, General Infirmary at Leeds, Great George Street, Leeds LS1 3EX, UK;* [2]*Diagnostic Development Unit, Department of Chemical Pathology, University of Leeds, Leeds LS2 9JT, UK.*

**Summary** This study was undertaken to evaluate the role of a new tumour marker, CA-242, alone or in combination with CEA in the practical management of colorectal cancer patients after potentially curative resection. A cohort of 149 patients who had undergone 'curative' surgery was followed up according to an intensive protocol in order to detect recurrent disease. Over a median tumour marker follow-up period of 24 months there were 25 recurrences in 24 patients. Both CEA and CA-242 alone detected half the local recurrences. The sensitivity of CEA was 84% for distant or mixed recurrence compared with 64% for CA-242. An abnormality of either CEA or CA-242 enabled detection of five out of six local recurrences and 17 out of 19 distant or mixed recurrences with a median lead time of 5 months for each marker. Both markers were elevated concurrently in only one local and 11 distant recurrences. While CA-242 alone is not superior to CEA, their combined use (either abnormal) has a high sensitivity (88%), specificity (78%) and negative predictive value (97%); this may be useful in reducing unnecessary investigations in follow-up programmes and as a guide to the initiation of further treatment for recurrent disease.

In the management of colorectal cancer operative findings, pathological stage and preoperative carcinoembryonic antigen (CEA) levels are strong prognostic indicators that are a guide to the likelihood of cure (Dukes & Bussey, 1958; Wanebo *et al.*, 1978). However, following potentially curative surgery there is a period of uncertainty as to whether the operation has cured the cancer in an individual patient. Treatment failure will usually become apparent during the first 2-3 years after surgery (Aldridge *et al.*, 1986; Sugarbaker *et al.*, 1987).

The precise post-operative surveillance procedures and their frequency vary but are based on clinical assessment, endoscopy, ultrasound and computerised tomography (CT) depending on the site of primary tumour. Many clinicians will include the measurement of CEA as an essential investigation in the detection of asymptomatic recurrence of colorectal cancer. Those surgeons who advocate the use of second-look surgery advise that CEA should be measured every 6-8 weeks so that a suspicious rising level can be identified as early as possible (Staab *et al.*, 1985; Minton & Chevinsky, 1989); for others 3 monthly CEA testing during the high-risk period of the first 2 years after resection tends to be the rule with a reduction in frequency of testing thereafter (Hine & Dykes, 1984).

It is evident that while CEA monitoring during the follow-up of patients after potentially curative surgery is valuable it lacks the sensitivity and specificity to be an infallible guide to the patient's status (Northover, 1986). Metastatic disease or local recurrence may produce symptoms or signs without a concurrent rise in CEA.

During recent years new generations of markers for gastrointestinal cancer, based on mucins, have become available as commercial test kits. They include CA-50 (Holmgren *et al.*, 1984), CA-19-9 (Del Villano *et al.*, 1983), CA-195 (Barghava *et al.*, 1987) and CA-242 (Nilsson *et al.*, 1992), all of which have been shown to be valuable in pancreatic cancer (Kuusela *et al.*, 1991; Taylor *et al.*, 1992; Pasanen *et al.*, 1993) and have been suggested as markers in colorectal cancer. When used in combination with CEA, CA-19-9 (Quentmeier *et al.*, 1987) and CA-195 (Sagar *et al.*, 1991; Ruggeri *et al.*, 1993) have been shown to provide a gain of positivity in colorectal cancer. The recent studies of CA-242 showed that it has a higher sensitivity than CA-50 in primary colorectal cancer and a low false positivity in benign liver disease (Kuusela *et al.*, 1991; Nilsson *et al.*, 1992). In primary colorectal cancer, additional use of CA-242 improves the diagnostic sensitivity of CEA alone (though it still remains limited) (Roberts *et al.*, 1992), and CA-242 has also been shown to complement CEA monitoring of patients receiving chemotherapy for liver metastases from colorectal cancer (Ward *et al.*, 1993). In this paper we report an evaluation of the combination of CEA and CA-242 in the post-operative monitoring of patients with colorectal cancer after curative surgery to assess the clinical utility of these markers in a well-defined group of patients.

Correspondence: N.R. Hall, Imperial Cancer Research Fund, Genetic Epidemiology Laboratory, St James's University Hospital, Beckett Street, Leeds LS9 7TF, UK.
Received 22 December 1993; and in revised form 15 April 1994.

### Patients and methods

We studied a cohort of patients who underwent laparotomy for colorectal carcinoma under the care of one surgeon (P.J.F.) between 1987 and 1991. Of the 246 consecutive patients treated in this period, 58 underwent a palliative procedure and are excluded: 22 of these had macroscopic residual disease remaining and 36 had evident metastatic disease at surgery. Thirty-nine further patients are excluded owing to early death preventing tumour marker follow-up or insufficient follow-up data. This leaves 149 patients who form the study group. All of these fulfil the following entry criteria for the study: 'curative' resection (surgical excision of all macroscopic disease), measurement of tumour markers on three or more occasions post-operatively and follow-up to recurrence, death or at least 6 months after the last tumour marker estimation included in the study. There were 68 males and 81 females with a median age of 70 years (range 37-87) and 72 years (range 42-86) respectively. Tumour site and Dukes' staging are shown in Table I.

Patients underwent a standard post-operative surveillance protocol involving outpatient attendance for clinical examination, faecal occult blood testing and tumour marker estimation every 3 months for the first 2 years, then 6 monthly. Liver ultrasound was performed every 6 months for the first 2 years, and for rectal tumours a pelvic CT scan was carried out annually. Colonoscopy was performed at 1 and 3 years after the operation, the first examination being earlier if a good-quality double-contrast barium enema had not been obtained prior to surgery. Patients were also investigated if there was a clinical suspicion of recurrent disease. The median follow-up period of tumour marker estimation was

**Table I** Tumour site and Dukes' staging

| *Dukes' stage* | *Right colon* | *Left colon* | *Rectum* | *Total (%)* |
|---|---|---|---|---|
| | *Tumour site*[a] | | | |
| A | 3 | 9 | 16 | 28 (19) |
| B | 29 | 25 | 24 | 78 (52) |
| C | 10 | 11 | 20 | 41 (28) |
| D | 0 | 2 | 0 | 2 (1) |
| Totals | 42 | 47 | 60 | 149 |

[a]Right colon, caecum to transverse colon; left colon, splenic flexure to rectosigmoid junction.

24 months and the clinical follow-up was for a median of 34 months.

CEA was measured by Hybritech two-site immunoradiometric Tandem CEA-RIA kits (Nottingham, UK) and CA-242 was measured using a dissociation-enhanced lanthanide fluoroimmunoassay (DELFIA, Wallac Oy, Turku, Finland). The upper limits of normal for these assays in our laboratory are 3 ng $ml^{-1}$ for CEA and 20 units $ml^{-1}$ for CA-242. Tumour marker levels were considered to be abnormal as follows: a single CEA level over 10 ng $ml^{-1}$ or three successively rising levels over 3 ng $ml^{-1}$; a single CA-242 over 40 units $ml^{-1}$ or three successively rising values over 20 units $ml^{-1}$.

### Results

### *Recurrent disease*

During follow-up there were 25 recurrences in 24 patients (16% of the cohort); one patient had an umbilical recurrence excised and later developed further abdominal wall recurrence and liver metastases. The sites of recurrence are shown in Figure 1 and the distribution according to Dukes' stage is tabulated in Table II. Recurrent disease occurred in three of the patients with Dukes' A tumours: one developed gross para-aortic lymphadenopathy but no evidence of liver metastasis, while the other two developed metastases in the lung, one also with liver secondaries. In the last two, histological inspection of the excised specimen demonstrated tumour permeation into vascular clefts within the muscularis propria. Both patients with Dukes' D tumours developed recurrence at the site of the distant disease excised at the original operation (the liver in one patient and the pelvis in the other). Twelve of the patients with rectal cancer developed recurrent disease (three locoregional only, one combined local and distant and eight distant disease only). The 13 recurrences in 12 patients with colonic primaries were as follows: three locoregional only, three mixed locoregional and distant and seven distant only. Median time to first recurrence was 17.5 months (range 8–23) for local only recurrences and 14.5 months (range 6–32) for distant or mixed local and distant recurrences.

One patient with pelvic recurrence was treated with radiotherapy and one patient with liver metastasis underwent a course of chemotherapy. Two patients had curative excision of umbilical recurrence. Three patients had elective second-look surgery and a fourth underwent emergency laparotomy for small bowel obstruction: all had unresectable disease. The only patient with a solitary liver metastasis declined further surgery. The remaining patients with recurrent or metastatic disease were treated symptomatically.

### *Tumour marker abnormalities*

During the study period markers were abnormal, as defined above, in 49 patients. Two strategies for the use of CEA and CA-242 in conjunction were adopted, with a positive test being counted either (a) when either marker is abnormal or (b) when both are abnormal. One or other marker was abnormal in 27 of 125 (22%) patients with no recurrence,

**Table II** Sites of recurrence according to Dukes' stage at presentation

| *Dukes' stage* | *None* | *Local/regional* | *Distant/mixed* | *Recurrence (%)* |
|---|---|---|---|---|
| | | *Recurrence* | | |
| A | 25 | 0 | 3 | 11 |
| B | 67 | 4 | 7 | 14 |
| C | 33 | 2 | 6 | 20 |
| D | 0 | 0 | 2 | 100 |
| Totals | 125 | 6 | 18 | |

**Figure 1** Sites of 25 recurrences in 24 patients.

five of six (83%) patients with local recurrence and in 17 of 18 (89%) patients (18 of 19 recurrences) with distant or mixed local and distant recurrence (Table III). Both markers were elevated in only one patient with local recurrence and 11 of the patients who developed distant or mixed recurrence.

CEA became abnormal in all patients who developed recurrent disease, either before or after recurrence had been diagnosed. In 17 the abnormality preceded clinical, histological or radiological confirmation of recurrent disease with a median lead time of 5 months (range 1–15). In two patients recurrence and CEA abnormality coincided. CA-242 did not rise to fulfil the criteria of abnormality in two patients with local disease and three patients with distant disease. In 14 it preceded confirmation of recurrence with a median lead time of 5 months (range 1–18).

The parameters quantifying accuracy of CEA and CA-242 alone or combined in the detection of recurrent (local and/or distant) colorectal carcinoma are shown in Table IV. CA-242 had a lower sensitivity than CEA (60% *vs* 76%) for recurrent disease. Specificity and positive and negative predictive values were each very similar for CEA and CA-242. Use of the 'either abnormal' strategy leads to a high sensitivity of 88%, a dimunution of specificity to 78% while the false-negative rate (1 – negative predictive value) falls to only 3%. For the 'both abnormal' approach the sensitivity falls to only 48% with a rise in specificity, positive predictive value and accuracy.

**Table III** CEA and CA-242 abnormalities according to presence and site of recurrent colorectal carcinoma

| Tumour marker | None (n = 125) | Recurrence Local/regional (n = 6) | Distant/mixed (n = 19, 18 patients) |
|---|---|---|---|
| CEA abnormality[a] | | | |
| Isolated | 6 | 3 | 14 |
| Rising | 14 | 2 | 12 |
| Either | 17 (14%) | 3 (50%) | 16 (84%) |
| CA-242 abnormality[a] | | | |
| Isolated | 13 | 3 | 12 |
| Rising | 8 | 3 | 5 |
| Either | 16 (13%) | 3 (50%) | 12 (63%) |
| CEA and CA-242 abnormal | 6 (5%) | 1 (17%) | 11 (58%) |
| CEA or CA-242 abnormal | 27 (22%) | 5 (83%) | 17 (89%) |

[a]Definitions of tumour marker abnormalities: CEA, isolated value $>10$ ng $ml^{-1}$, three rising values over 3 ng $ml^{-1}$; CA-242, isolated value $>40$ U $ml^{-1}$, three rising values over 20 U $ml^{-1}$.

**Table IV** Five measurements of accuracy for CEA and CA-242 alone or in combination in the detection of recurrent colorectal carcinoma

| | CEA alone (%) | CA-242 alone (%) | CEA and CA-242 combined (%) Both abnormal | Either abnormal |
|---|---|---|---|---|
| Sensitivity | 76 | 60 | 48 | 88 |
| Specificity | 86 | 87 | 95 | 78 |
| Positive predictive value | 53 | 49 | 67 | 45 |
| Negative predictive value | 95 | 92 | 90 | 97 |
| False-negative rate | 5 | 8 | 10 | 3 |
| Overall accuracy | 85 | 83 | 87 | 80 |

### Discussion

Reports of tumour marker abnormalities depend critically on the criteria used to define the abnormality. For single measurements (e.g. preoperative levels) it is possible to compare the specificity and sensitivity of two tests independently of the cut-off level used to define the abnormality by using receiver operating characteristic (ROC) curve analysis (Pasanen *et al.*, 1993; Zweig & Campbell, 1993). It may also facilitate the choice of appropriate cut-off level. The nature of serial tumour marker estimations, however, is such that a level may fluctuate, sometimes excessively, often peaking above the reference range before returning below it in the absence of any recurrent cancer. By contrast, a steadily rising level is much more suggestive of the presence of malignant tissue, even if the absolute value may not be greatly elevated. Sometimes the growth of tumour is rapid and a suddenly high level may be the first indication of recurrent disease. Different authors have adopted differing solutions to this problem, some relying on a single elevated level (Beart & O'Connell, 1983; Hine & Dykes, 1984), some on a trend (Staab *et al.*, 1985; Sugarbaker *et al.*, 1987), and others using more complicated analytical methods (Martin *et al.*, 1977). This issue was addressed specifically by Denstman *et al.* (1986), but although some form of slope analysis was recommended in preference to an absolute cut-off value no firm guidelines could be offered. In this study we adopted a definition combining the two decision methods (see section on tumour marker estimation) with emphasis on simplicity and ease of use in a clinical setting.

Carcinoembryonic antigen was first described by Gold and Freedman (1965) and for colorectal carcinoma it remains, nearly 30 years later, the gold standard by which new markers are judged. The current study confirms the reliability of CEA levels as a guide to recurrent disease, though there remains the problem, experienced by others (Northover, 1986), that in 14% of the patients without recurrence there was also an abnormality of CEA (Table III). CA-242 performed very similarly to CEA though with a reduced sensitivity. Neither marker was as good in the detection of local recurrence as distant, though the numbers are too small to make firm conclusions about which test was superior for local disease detection. Although the majority of patients with recurrence had concomitant rise of CEA and CA-242, there were instances in which one marker remained normal while the other rose; thus the use of the two in combination (either abnormal) increased the sensitivity to 88% (Table IV). This is achieved at the expense of reduced specificity and an increased rate of false positivity. A strategy requiring both markers to be abnormal, while having a high specificity for the detection of recurrence, has an unacceptably low sensitivity of only 48% and is unlikely to be of practical value.

A major drawback of the early diagnosis of recurrent colorectal cancer is the present lack of an effective treatment for the majority of patients. For some, resection of a local recurrence can be curative, and even where there are distant metastases (if few in number) resection may improve prognosis. For the majority of patients, however, early diagnosis may only lengthen the period of anxiety before death. In our cohort only 2 of the 16 patients with recurrent disease underwent potentially curative surgery (both had umbilical disease resected) and one of these remains well 2 years later with no sign of further disease. Neither patient had elevated tumour marker levels at diagnosis of recurrence. To take the nihilistic view that early diagnosis is of no value, however, would be to negate future advances in treatment and the positive benefits to patients of knowing their destiny.

Initial enthusiasm that CEA-assisted detection of recurrent disease might improve the ability of second-look radical surgery to provide a lasting cure has been tempered by the overall results from some studies (Beart & O'Connell, 1983). A multicentre trial in the UK addressing this issue is nearing completion. Previous reports show that only about half the patients who undergo second-look surgery have resectable disease, though the proportion who have further potentially curative surgery is higher in patients with asymptomatic recurrence detected by CEA; survival in the latter group is also improved (Minton et al., 1985; Staab et al., 1985; Quentmeier et al., 1990).

The role of tumour markers extends wider than as an indicator for further surgery. Firstly, the follow-up of colorectal cancer by regular haematological, biochemical and radiological investigation is costly and the majority of investigations are normal (Sugarbaker et al., 1987). Cost-effectiveness may be improved by the use of CEA (or other tumour marker) directed investigation (Wanebo et al., 1989). Our results show that a normal CEA or CA-242 is rarely seen in the presence of recurrent disease, whereas tumour can eventually be demonstrated in about half the patients in whom either tumour marker is abnormal (positive predictive value 49–51%). This rather low positive predictive value may be considered acceptable given the low false-negative rate. It is clear that CA-242 alone is inferior to CEA as a tumour marker, though it may serve to complement CEA in the follow-up after curative resection for colorectal cancer. In a setting in which investigations were marker directed (as opposed to routine) there might be a significant advantage in having a dual tumour marker assay to give the required high sensitivity, accepting that there may also be an increased negative investigation rate. We are currently examining a larger cohort of patients to evaluate such a policy.

Secondly, specificity of production of the CEA and CA-242 antigens by malignant tissue has opened the possibility for targeting other molecules at the cancer itself by coupling them with antibodies to these epitopes. For example, *Pseudomonas* exotoxin coupled to C242 (the antibody that recognises the antigen CA-242) has been effective against a human colorectal cancer xenograft in nude mice (Debinski et al., 1992).

A third, and as yet unexplored, avenue relates to the use of newer therapies directed against recurrent disease. If second-look surgery has failed to gain general support owing to the relatively small proportion of patients who derive benefit, then perhaps a low-toxicity chemotherapy regimen may prove to be advantageous in patients who develop elevated tumour markers. Currently the American National Institutes of Health (1990) recommends adjuvant chemotherapy (Moertel et al., 1990) for a subgroup of patients with colorectal cancer in the knowledge that even without chemotherapy half may have a lasting cure. On the basis that chemotherapeutic agents are most effective in the presence of minimal disease there is an argument for treating patients who have a rise in tumour marker levels even before recurrence is demonstrated radiologically or clinically. We have confirmed a median lead time of raised tumour markers of 5 months before recurrence is detectable by other methods of investigation, and treatment at such an early stage is likely to maximise the potential benefit. Unfortunately, this would also mean treating about half the patients unnecessarily, but that may be acceptable provided the toxicity was minimised. As chemotherapeutic regimens improve, the importance of tumour markers as an indication for therapy will probably increase.

For the reasons above, coupled with the results of this study, we feel that an approach using tumour markers in combination is likely to improve their value in the follow-up of colorectal carcinoma.

N.R. Hall is in receipt of an Imperial Cancer Research Fund Clinical Fellowship.

**References**

ALDRIDGE, M.C., PHILLIPS, R.K.S., HITTINGER, R., FRY, J.S. & FIELDING, L.P. (1986). Influence of tumour site on presentation, management and subsequent outcome in large bowel cancer. *Br. J. Surg.*, **73**, 663–670.

BARGHAVA, A., PETRELLI, N.J., GAUR, P., BROWN, W. & FITZPATRICK, J. (1987). Circulating CA 195 in colorectal cancer. *J. Tumor Marker Oncol.*, **2**, 319–327.

BEART, R.W. & O'CONNELL, M.J. (1983). Postoperative follow-up of patients with carcinoma of the colon. *Mayo Clin. Proc.*, **58**, 361–363.

DEBINSKI, W., KARLSSON, B., LINDHOLM, L., SIEGALL, C.B., WILINGHAM, M.C., FITZGERALD, D. & PASTAN, I. (1992). Monoclonal antibody C242-*Pseudomonas* exotoxin A: a specific and potent immunotoxin with antitumor activity on a human colon cancer xenograft in nude mice. *J. Clin. Invest.*, **90**, 405–411.

DEL VILLANO, B.C., BRENNAN, S., BROCK, P., BUCHER, C., LIU, V., MCCURE, M., RAKE, B., SPACE, S., WESTRICK, B., SCHOEMAKER, H. & ZURAWSKI, Jr., V.R. (1983). Radioimmunometric assay for a monoclonal antibody-defined tumor marker, CA 19-9. *Clin. Chem.*, **29**, 549–552.

DENSTMAN, F., ROSEN, L., KHUBCHANDANI, I.T., SHEETS, J.A., STASIK, J.J. & REITHER, R.D. (1986). Comparing predictive decision rules in postoperative CEA monitoring. *Cancer*, **58**, 2089–2095.

DUKES, C.E. & BUSSEY, H.J.R. (1958). The spread of rectal cancer and its effect on prognosis. *Br. J. Cancer*, **12**, 309–320.

GOLD, P. & FREEDMAN, S.O. (1965). Demonstration of tumour specific antigens in human colonic carcinomata by immunological tolerance and adsorption techniques. *J. Exp. Med.*, **121**, 439–462.

HINE, K.R. & DYKES, P.W. (1984). Serum CEA testing in the post-operative surveillance of colorectal carcinoma. *Br. J. Cancer*, **49**, 689–693.

HOLMGREN, J., LINDHOLM, L., PERSSON, B., LAGERGÅRD, T., NILSSON, O., SVENNERHOLM, L., RUDENSTAM, C.-M., UNSGAARD, B., YNGVASON, F., PETTERSSON, S. & KILLANDER, A.F. (1984). Detection by monoclonal antibody of carbohydrate antigen CA 50 in serum of patients with carcinoma. *Br. Med. J.*, **288**, 1479–1482.

KUUSELA, P., HAGLUND, C. & ROBERTS, P.J. (1991). Comparison of a new tumour marker CA 242 with CA 19-9, CA 50 and carcinoembryonic antigen (CEA) in digestive tract diseases. *Br. J. Cancer*, **63**, 636–640.

MARTIN, E.W., JAMES, K.K., HURTUBISE, P.E., CATALANO, P. & MINTON, J.P. (1977). The use of CEA as an early indicator for gastrointestinal tumor recurrence and second-look procedures. *Cancer*, **39**, 440–446.

MINTON, J.P. & CHEVINSKY, A.H. (1989). CEA directed second-look surgery for colon and rectal cancer. *Ann. Chirurg. Gynaecol.*, **78**, 32.

MINTON, J.P., HOEHN, J.L., GERBER, D.M., HORSLEY, J.S., CONNOLLY, D.P., SALWAN, F., FLETCHER, W.S., CRUZ, A.B., GATCHELL, F.G., OVIEDO, M., MEYER, K.K., LEFFALL, L.D., BERK, R.S., STEWART, P.A. & KURUCZ, S.E. (1985). Results of a 400-patient carcinoembryonic antigen second-look colorectal cancer study. *Cancer*, **55**, 1284–1290.

MOERTEL, C.G., FLEMING, T.R., MACDONALD, J.S., HALLER, D.G., LAURIE, J.A., GOODMAN, P.J., UNGERLEIDER, J.S., EMERSON, W.A., TORMEY, D.C., GLICK, J.H., VEEDER, M.H. & MAILLIARD, J.A. (1990). Levamisole and fluorouracil for adjuvant therapy of resected colon carcinoma. *N. Engl. J. Med.*, **322**, 352–358.

NIH CONSENSUS CONFERENCE (1990). Adjuvant therapy for patients with colon and rectal cancer. *JAMA*, **264**, 1444–1450.

NILSSON, O., JOHANSSON, C., GLIMELIUS, B., PERSSON, B., NØRGAARD-PEDERSEN, B., ANDRÉN-SANDBERG, Å. & LINDHOLM, L. (1992). Sensitivity and specificity of CA242 in gastrointestinal cancer. A comparison with CEA, CA50 and CA 19-9. *Br. J. Cancer*, **65**, 215–221.

NORTHOVER, J. (1986). Carcinoembryonic antigen and recurrent colorectal cancer. *Gut*, **27**, 117–122.

PASANEN, P.A., ESKELINEN, M., PARTANEN, K., PIKKARAINEN, P., PENTTILÄ, I. & ALHAVA, E. (1993). Receiver operating characteristic (ROC) curve analysis of the tumour markers CEA, CA 50 and CA 242 in pancreatic cancer; results from a prospective study. *Br. J. Cancer*, **67**, 852–855.

QUENTMEIER, A., SCHLAG, P., GIESEN, H.P. & SCHMIDT-GAYK, H. (1987). Evaluation of Ca 12-5 as a tumor marker for gastric and colo-rectal cancer in comparison with CEA and Ca 19-9. *Eur. J. Surg. Oncol.*, **13**, 197–201.

QUENTMEIER, A., SCHLAG, P., SMOK, M. & HERFARTH, C. (1990). Re-operation for recurrent colorectal cancer: the importance of early diagnosis for resectability and survival. *Eur. J. Surg. Oncol.*, **16**, 319–325.

ROBERTS, P.J., KUUSELA, P., CARPELAN-HOLMSTRÖM, M., HAGLUND, C. (1992). Value of different tumor markers in colorectal cancer. In *Tumour Associated Antigens, Oncogenes, Receptors, Cytokines in Tumor Diagnosis and Therapy at the Beginning of the Nineties*, Klapdor, R. (ed.) pp. 30–32. W. Zuckschwerdt: München.

RUGGERI, G., BELLOLI, S., MONTINARI, F., SEREGNI, E., BOMBADIERI, E., ALBERTINI, A. (1993). CA 195 as a tumor marker in patients with gastrointestinal cancer. In *Updating on Tumor Markers in Tissues and Biological Fluids*, Ballesta, A. M., Torre, G.C., Bombadieri, E., Gion, M., Molina, R. (eds) pp. 545–553. Minerva Medica: Turin.

SAGAR, P.M., TAYLOR, O.M., COOPER, E.H., BENSON, E.A., MCMAHON, M.J. & FINAN, P.J. (1991). The tumour marker CA 195 in colorectal and pancreatic cancer. *Int. J. Biol. Markers*, **6**, 241–246.

STAAB, H.J., ANDERER, F.A., STUMPF, E., HORNUNG, A., FISCHER, R. & KIENINGER, G. (1985). Eighty-four potential second-look operations based on sequential carcinoembryonic antigen determinations and clinical investigations in patients with recurrent gastrointestinal cancer. *Am. J. Surg.*, **149**, 198–204.

SUGARBAKER, P.H., GIANOLA, F.J., DWYER, A. & NEUMAN, N.R. (1987). A simplified plan for follow-up of patients with colon and rectal cancer supported by prospective studies of laboratory and radiologic test results. *Surgery*, **102**, 79–87.

TAYLOR, O.M., COOPER, E.H., BENSON, E.A. & MCMAHON, M.J. (1992). The prognostic value of the tumour markers CA 195 and CEA in patients with adenocarcinoma of the pancreas. *Eur. J. Surg. Oncol.*, **18**, 508–513.

WANEBO, H.J., RAO, B., PINSKY, C.M., HOFFMAN, R.G., STEARNS, M., SCHWARTZ, M.K. & OETTGEN, H.F. (1978). Preoperative carcinoembryonic antigen level as a prognostic indicator in colorectal cancer. *N. Engl. J. Med.*, **299**, 448–451.

WANEBO, H.J., LLANERAS, M., MARTIN, T. & KAISER, D. (1989). Prospective monitoring for carcinoma of colon and rectum after surgical resection. *Surg. Gynecol. Obstet.*, **169**, 479–487.

WARD, U., PRIMROSE, J.N., FINAN, P.J., PERREN, T.J., SELBY, P., PURVES, D.A. & COOPER, E.H. (1993). The use of tumour markers CEA, CA-195 and CA-242 in evaluating the response to chemotherapy in patients with advanced colorectal cancer. *Br. J. Cancer*, **67**, 1132–1135.

ZWEIG, M.H. & CAMPBELL, G. (1993). Receiver-operating characteristic (ROC) plots: a fundamental evaluation tool in clinical medicine. *Clin. Chem.*, **39**, 561–577.